# *Chryseobacterium gleum* in a man with prostatectomy in Senegal: a case report and review of the literature

**DOI:** 10.1186/s13256-017-1269-4

**Published:** 2017-04-24

**Authors:** O. Arouna, F. Deluca, M. Camara, B. Fall, B. Fall, A. Ba Diallo, J. D. Docquier, S. Mboup

**Affiliations:** 1 Laboratoire de Bactériologie-Virologie, CHNU Aristide Le Dantec, Université Cheikh Anta Diop, BP 7325 Dakar, Senegal; 2 Dipartimento di Biotcnologie Mediche, Università degli Studi di Siena, Policlinico “Le Scotte”, V lotto, II piano, Viale Bracci, 16, 53100 Siena (SI), Italy; 3 Service d’Urologie, CHNU Aristide Le Dantec, BP 7325 Dakar, Senegal; 4 Fédérations des laboratoires, Hôpital Principal de Dakar, 1, Avenue Nelson Mandela, Dakar, Senegal

**Keywords:** *Chryseobacterium gleum* case report, Urinary tract infection, Metallo-β-lactamase, CGB-1, resistance

## Abstract

**Background:**

Here we report a rare case of a urinary tract infection due to *Chryseobacterium gleum*. This widely distributed Gram-negative bacillus is an uncommon human pathogen and is typically associated with health care settings.

**Case presentation:**

We describe a case of urinary tract infection caused by *Chryseobacterium gleum* in a 68-year-old man of Wolof ethnicity (an ethnic group in Senegal, West Africa) who presented to our Department of Urology in a university teaching hospital (Hôpital Aristide Le Dantec) in Dakar, Senegal, 1 month after prostatectomy. The strain isolated from a urine sample was identified as *Chryseobacterium gleum* by mass spectrometry (Vitek matrix-assisted laser desorption/ionization, time-of-flight, bioMérieux) and confirmed by 16S ribosomal ribonucleic acid sequencing. The organism was resistant to a wide range of antibiotics, including carbapenem, due to a resident metallo-β-lactamase gene that shared 99% of amino-acid identity with Chryseobacterium gleum class B enzym.

**Conclusions:**

Infection by *Chryseobacterium gleum* is infrequent, and no such case has been previously reported in Africa. Despite its low virulence, *Chryseobacterium gleum* should be considered a potential opportunistic and emerging pathogen. Further studies on the epidemiology, pathogenicity, and resistance mechanisms of *Chryseobacterium gleum* are needed for better diagnosis and management.

## Background

The genus *Chryseobacterium* (formerly *Flavobacterium*) [1], whose type species is *Chryseobacterium gleum* [2] and belongs to the family *Flavobacteriaceae* (phylum Bacteroidetes), represents a group of Gram-negative, non-fermenting, catalase-positive, and indole-positive aerobic bacilli. *Chryseobacterium* species are uncommon human pathogens, and most cases are nosocomial and are often associated with immunosuppression or indwelling devices. *Chryseobacterium* is typically found in soil, water, plants, and food products and can survive in hospital environments, chlorinated water, and wet surfaces, all of which serve as potential reservoirs of infection. A literature search revealed only a few published cases in countries including Hungary, India, Croatia, and Saudi Arabia [3–6]. In Senegal, two cases of meningitis due to *Flavobacterium meningosepticum* were diagnosed in the late 1970s [7] as well as one case of *Chryseobacterium indologenes* [8]. However, human *C. gleum* infection has not been previously reported in Africa.

Here, we report the first case of *C. gleum* infection occurring in Africa and provide a review of similar cases worldwide (Table 1).

**Table 1:** Case reports of infection by *Chryseobacterium gleum*

Author	Country	Sex^a^/^b^Age	Site	Outcome	Factors	Antibiotic treatment
Virok *et al*. (2014) [3]	Hungary	Newborns	Stomach content	Cured	Respiratory tract involvement	Ciprofloxacin
Garg *et al*. (2015) [18]	India	M/48	Urine	Cured	Pyonephrosis	Tetracycline
Dijana *et al*. (2015) [5]	Croatia	F/35	Blood/Tracheal aspirate	Cured	Hepatic lesion/malnutrition	Piperacillin/Tazobactam
Ramya *et al*. (2015) [4]	India	M/62	Urine	Cured	Renal calculi/Hydronephrosis	Piperacillin/Tazobactam
Baha *et al*. (2016) [6]	Saudi Arabia	Newborn	Endotracheal aspirate	Cured	Nephrotic syndrome	Levofloxacin
Our case (2016)	Senegal	M/68	Urine	Cured	Prostatectomy	Ciprofloxacin

^a^ *M* male, *F* female

^b^ *Y* year

## Case presentation

A 68-year-old man of Wolof ethnicity (an ethnic group in Senegal, West Africa) presented to our Department of Urology with severe dehydration and general deterioration of condition 1 month after prostatectomy via transurethral resection performed at a regional hospital. At hospital admittance, a clinical examination found a high blood pressure (180/90 mmHg) and a fever of 38.5 °C. Blood tests found leukocytosis with a level of 17,100/mL white blood cells (WBC), anemia with a level of 10 g/dL, and creatinine with a level of 513.3 μmol/L. Serum electrolytes showed hyponatremia with a level of 111 mEq/L, hypokalemia with a level of 3.3 mEq/L, and hypochloremia with a level of 77 mEq/L. At presentation, he had an indwelling urinary catheter draining urine incorrectly that required a urine bacteriology examination.

Treatment consisted of fluid and electrolyte replacement, daily dressing of the wound, and empiric antibiotic therapy with amikacin. This drug was chosen because of the severity of the infection and because our patient had previously received treatment with fluoroquinolone. Seven days after hospitalization, the outcome was favorable with a clean surgical wound and normalization of serum potassium and chloride levels. However, hyponatremia persisted at 119 mEq/L, and his serum creatinine level was 150.45 μmol/L. Removal of the urinary catheter was performed on day 7 of hospitalization, and he returned home the same day.

The cloudy urine collected for bacteriology examination at hospitalization was inoculated on a cysteine lactose electrolyte deficient (CLED) agar plate according to the usual techniques for medical bacteriology. Direct examination showed a rich bacterial flora, many white cells, and Gram-negative bacilli by Gram staining. The CLED agar grew yellow-colored 1 to 2 mm circular colonies (>10^6^ CFU/mL) with regular margins.

After culture, *Chryseobacterium* species was suspected because of the yellow-colored colonies due to flexirubin production. Identification using the Appareil et Procédés d’Identification 20 NE identification system (bioMérieux, France) gave *Myroides* species and *C. indologenes* after, respectively, 24 hours and 48 hours of incubation (Table 2).

**Table 2:** Identification of the strain using Appareil et Procédés d’Identification 20 NE (V7.0)

	%ID		T	
	24 H	48H	24H	48H
Significant taxa				
*Myroides* species	61.2	–	0.67	–
*Chryseobacterium indologenes*	34.2	99.9	0.84	0.42
Next taxon				
*Bergeyella zoohelcum*	4.4	–	0.5	–
*Shewanella putrefaciens* group		0.1		0.0

*24 H* 24 hours of incubation, *48 H* 48 hours of incubation, *%ID* identification percentage (proximity relative to the different taxa in the database. It is determined whether the observed profile is closer to a particular taxon), *T* typicality (the most typical profile is the one that has no tests against the identification compared with percentages in the database for that taxon)

However, mass spectrometry (Vitek MS matrix-assisted laser desorption/ionization, time-of-flight, bioMérieux) successfully identified *C. gleum* and the result was confirmed using 16S ribosomal ribonucleic acid (rRNA) sequencing.

In this case, *C. gleum* was confirmed as the causative agent for our patient’s urinary tract infection as evidenced from the culture reports. Confluent growth of the organism in pure form from urine helped to rule out contamination.

Antimicrobial susceptibility testing using disc diffusion and E-tests (bioMérieux) for the minimum inhibitory concentration (MIC) using *Pseudomonas aeruginosa* ATCC 27853 and *Escherichia coli* ATCC 25922 for internal quality control was completed and interpreted per the Comité d’Antibiogramme de la Société Française de Microbiologie (CA-SFM) recommendations of 2015. The isolated strain was susceptible to piperacillin (MIC = 6 μg/mL), ceftazidime (MIC = 0.75 μg/mL), cefepime (MIC = 0.125 μg/mL), ciprofloxacin (MIC = 0.25 μg/mL), imipenem (MIC = 2 μg/mL), and resistant to meropenem (MIC = 8 μg/mL), aztreonam (MIC ≥256 μg/mL), ticarcillin (MIC ≥256 μg/mL), ticarcillin-clavulanic acid (MIC = 64 μg/mL), and cefotaxime (MIC >32 μg/mL; Table 3).

**Table 3:** *In vitro* antimicrobial susceptibilities of the strain, and interpretation according to Comité d’Antibiogramme de la Société Française de Microbiologie 2015 recommendations, using *Pseudomonas aeruginosa* ATCC 27853 and *Escherichia coli* ATCC 25922 as quality control

Antibiotics	MIC	IZD	Breakpoint^a^	S	Categorization
	(μg/mL)	(mm)	(μg/mL)	(mm)	
Ticarcillin	≥256	0	≤16	≥18	Resistant
Ticarcillin + AC	64	8	≤16	≥18	Resistant
Piperacillin	6	26	≤16	≥18	Susceptible
Ceftazidime	0.75	22	≤8	≥16	Susceptible
Cefotaxime	>32	8	-	-	Resistant
Cefepime	0.125	37	≤8	≥19	Susceptible
Aztreonam	≥256	0	≤1	≥50	Resistant
Imipenem	2	25	≤4	≥20	Susceptible
Meropenem	8	15	≤2	≥18	Resistant
Ciprofloxacin	0.25	30	≤0.5	≥25	Susceptible
Co-trimoxazole	0.094	28	-	-	Susceptible

*-* cefotaxime breakpoint not given, *AC* clavulanic acid, *IZD* inhibition zone diameter, *MIC* minimum inhibitory concentration, *S* Susceptibility

^a^Antimicrobial susceptibilities interpretation was done according to Comité d’Antibiogramme de la Société Française de Microbiologie 2015 recommendations using *Pseudomonas* species as reference

**Fig. 1:**
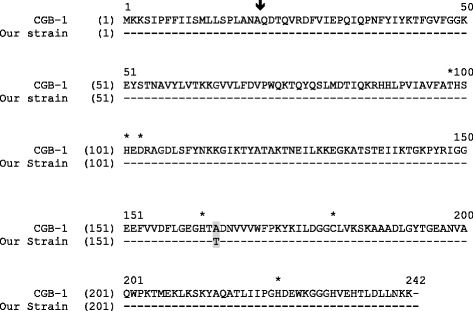
Comparison of the amino acid sequence of β-lactamase CGB-1 with our strain. *Broken lines* indicate identical amino acid residues. The *vertical arrow* indicates the putative cleavage site for the leader peptide of CGB-1. Amino acids that may be involved in the binding of Zinc (Zn^2+^) or/and water are indicated with a *star. Shading* corresponds to the single amino-acid mutation occurring in the gene of our strain

Due to the decreased susceptibility to carbapenem, a combined test with imipenem and ethylenediaminetetraacetic acid (EDTA) was positive, indicating the production of metallo-β-lactamase (MBL). To characterize the gene encoding MBL, polymerase chain reaction (PCR) was performed using genomic deoxyribonucleic acid (DNA) obtained by phenol–chloroform extraction, using the template primers CGB1_exp/fw (5′-GGGAATTCCATATGAAAAAAAGCATTCCGTTTTTTA) and CGB1_exp/rv (CGCGGATCCTTATTTTTTATTTAAAAGATCAAG) as described previously by Bellais and colleagues [9]. DNA sequence analysis of the 726-base pairs (bp) amplified fragments encoding a 242-amino acid preprotein showed 99% similarity with CGB-1 (Fig. 1).

## Discussion

*C. gleum* is an unusual human pathogen that has been reported as the cause of early neonatal respiratory infection in three neonates [3]. The most common species causing human disease is *F. meningosepticum* followed by *C. indologenes* and *C. gleum* [10]. Typically thought of as an organism of low virulence, *C. gleum* may cause serious infections, particularly among immunocompromised patients. Colonization of this organism in patients can occur via contaminated medical devices involving fluids, such as intubation tubes, respirators, humidifiers, and incubators for newborns, as previously reported [11]. Almost, all publications available in the literature refer to long hospital stays with prolonged antibiotic treatment (>14 days), oncological disease, and most of the patients had immunosuppression or indwelling devices [8–11]. A recent case published in 2016 from Saudi Arabia, reported *C. gleum* pneumonia in a 6-month-old baby with nephrotic syndrome [6]. The patient, intubated, was previously on multiple courses of antibiotics including meropenem, ceftriaxone, and vancomycin. Virok *et al*. reported in 2014 a case of early neonatal respiratory infection due to *C. gleum* in Hungary [3]. In Croatia, Dijana *et al*. published in 2015 a case of *C. gleum* infection in a patient with malnutrition and hepatic lesion [5]. The patient with respiratory insufficiency had developed septic shock, which necessitated intubation and mechanical ventilation. Our patient underwent a prostatectomy in a regional hospital with indwelling catheter; he had a urinary tract infection due to *C. gleum*, similar to an Indian case reported by Ramya *et al*. in 2015 [4].

Little is known about the virulence factor(s) of the members of *Chryseobacterium*, although reports have indicated that biofilm and protease production are important mechanisms involved in the virulence of *Elizabethkingia meningoseptica* (formerly *Chryseobacterium meningosepticum*) and *C. indologenes* [11]. Biofilm production by *C. indologenes* in central venous catheter-related bloodstream infection was recently noted [12]. *E. meningoseptica* isolates demonstrated 100% biofilm-forming ability in Luria-Bertani (LB) media, and increasing biofilm production is associated with the mortality rates of infected patients [13]. Lo and Chang performed a focused study on 14 *C. gleum* isolates [14], and the strains were epidemiologically unrelated and originated primarily from urine and sputum. Their study demonstrated that 40% of *C. gleum* isolates could form a biofilm. The biofilm-forming potential of *C. gleum* appeared to be much lower than that of *E. meningoseptica*. Therefore, it was suggested that *C. gleum* might be less virulent than *E. meningoseptica* and *C. indologenes*. In fact, we previously reported a fatal case of urinary tract infection due to *C. indologenes* in a woman with acute leukemia [8]. This outcome is in contrast to the present case, where the patient survived despite an advanced state of malnutrition.

Only 0.27% (50 of 18,569) of all processed non-fermenting Gram-negative bacilli (NFGNBs) and 0.03% (50 of 15,5811) of isolates collected by the SENTRY Antimicrobial Surveillance Program during the 5-year period from 1997 to 2001 were members of the genera *Chryseobacterium* or *Elizabethkingia* (formerly *Chryseobacterium*), with the most frequent organisms being *E. meningoseptica*, *C. indologenes*, and *C. gleum* [15]. All 50 isolates were obtained from hospitalized patients, among which only two isolates (4%) were identified as *C. gleum*. The present case is the first reported case of a urinary tract infection in Africa by *C. gleum*, although *C. indologenes* was previously reported in Burkina Faso in 2007 [16], in Senegal in 2014 [8], and in Tunisia in 2015 [17]. However, in Senegal, two cases of meningitis due to *F. meningosepticum* were diagnosed in the late 1970s [7].

There are few data available on antimicrobial susceptibility and no standardized breakpoints for *Chryseobacterium* species in CA-SFM guidelines due to the small number of clinical isolates. Thus, selecting an antimicrobial agent for the treatment of *Chryseobacterium* species infections remains difficult, especially because of chromosomal resistance to many antibiotics used for Gram-negative bacteria. According to the SENTRY Antimicrobial Surveillance Program, the antimicrobials most active against *Chryseobacterium* species are quinolones and sulfamethoxazole-trimethoprim (≥95% susceptibility) followed by piperacillin-tazobactam (90% susceptibility). Ciprofloxacin, cefepime, ceftazidime, piperacillin, and rifampicin showed reasonable activity (85% susceptibility), whereas aminoglycosides, other β-lactams, chloramphenicol, linezolid, and glycopeptides are not appropriate for treating infections caused by this organism [15]. This conclusion is in concordance with the drug susceptibility pattern of the strain isolated in Senegal. In fact, most *C. gleum* infections have been preferentially treated by newer generations of quinolones than by the combination of piperacillin-tazobactam (Table 1). However, the strain isolated in Saudi Arabia was resistant to piperacillin-tazobactam. In addition, it was previously published that *C. gleum* CIP 103039 carried at least two likely chromosome-encoded β-lactamases, Amber class A CGA-1 and Amber class B CGB-1, and was resistant to penicillins, cephalosporins, cefotaxime, and aztreonam, intermediate to carbapenems, and susceptible to piperacillin. Our strain showed the same drug susceptibility pattern as *C. gleum* CIP 103039 (Table 4). In contrast, the focused study of 14 *C. gleum* isolates conducted in Taiwan by Lo and Chang revealed that all isolates were resistant to piperacillin and imipenem [14].

**Table 4:** Minimum inhibitory concentrations of β-lactams for C*hryseobacterium gleum* CIP 103039, in comparison with our strain

MIC (μg/mL) for:		
Antibiotics	Our strain	*Chryseobacterium gleum* CIP 103039
Ticarcillin	≥256	256
Ticarcillin + AC	64	256
Piperacillin	6	2
Ceftazidime	0.75	4
Cefotaxime	>32	32
Cefepime	0.125	1
Aztreonam	≥256	>512
Imipenem	2	2
Meropenem	8	8

*AC* clavulanic acid, *MIC* minimum inhibitory concentration

## Conclusions

To the best of our knowledge, this is the first case of a *C. gleum* infection reported in Africa. Despite their low virulence, *Chryseobacterium* species should be considered a potential opportunistic pathogen that can cause infection in hospitalized patients. This report highlights the emergence of *Chryseobacterium* infections in Africa and emphasizes the importance of strengthening hospital hygiene measures as well as the necessity to survey environmental bacteria that could cause hospital-acquired infections.
